# A New Approach to Establish a Cell Line with Reduced Risk of Endogenous Retroviruses

**DOI:** 10.1371/journal.pone.0061530

**Published:** 2013-04-09

**Authors:** Aiko Fukuma, Rokusuke Yoshikawa, Takayuki Miyazawa, Jiro Yasuda

**Affiliations:** 1 Department of Emerging Infectious Diseases, Institute of Tropical Medicine (NEKKEN), Nagasaki University, Nagasaki, Japan; 2 Laboratory of Signal Transduction, Institute for Virus Research, Kyoto University, Kyoto, Japan; National Institute of Allergy and Infectious Diseases, United States of America

## Abstract

Endogenous retroviruses (ERVs) are integrated as DNA proviruses in the genomes of all mammalian species. Several ERVs are replication-competent and produced as fully infectious viruses from host cell. Thus, live-attenuated vaccines and biological substances have been prepared using the cell lines which may produce ERV. Indeed, we recently reported that several commercial live-attenuated vaccines for pets were contaminated with the infectious feline endogenous retrovirus, RD-114. In this study, to establish a cell line for vaccine manufacture with reduced risk of ERVs, we generated a cell line stably expressing human tetherin (Teth-CRFK cells). The release of infectious ERV from Teth-CRFK cells was suppressed to undetectable levels, while the production of parvovirus in Teth-CRFK cells was similar to that in parental CRFK cells. These observations suggest that Teth-CRFK cells will be useful as a cell line for the manufacture of live-attenuated vaccines or biological substances with reduced risk of ERV.

## Introduction

Endogenous retroviruses (ERVs) are integrated as DNA proviruses in the genomes of all mammalian species. Several ERVs are replication-competent and produced as fully infectious viruses from host cells, although most are inactivated by deletions and mutations with stop codons [1]. ERVs have also been reported to be produced constitutively or by chemical induction in some cell lines [2], [3]. Many live-attenuated vaccines and biological substances have been prepared using these cell lines.

Recently, we reported that several commercial live-attenuated vaccines for pets were contaminated with the infectious feline endogenous gammaretrovirus, RD-114 [4]–[7]. All domestic cats have an infectious RD-114 [2], [8], [9]. Several feline cell lines, such as Crandell–Rees feline kidney (CRFK) cells, produce RD-114 [2], [10], [11], and many live-attenuated vaccines for dogs and cats are produced using these feline cell lines.

RD-114 is a polytropic virus [11] and has the potential risk that interspecies transmission may induce unpredictable diseases, although the pathogenicity of RD-114 has not been demonstrated. However, it is very difficult to completely exclude the proviral DNA of RD-114 from cells, as ERVs are usually integrated into multiple loci in the host chromosomes. Therefore, it is necessary to develop a new approach to reduce the risk by regulating the production of infectious RD-114 from cells.

Tetherin (also referred to as BST-2, CD317, or HM1.24) was originally identified as a cellular restriction factor that blocks the release of HIV-1 in the absence of the viral accessory protein, Vpu [12], [13]. Subsequent studies have shown that tetherin also inhibits the release of other retroviruses including gammaretroviruses, filoviruses, arenaviruses, and herpesviruses [12], [14]–[23]. Recently, we found that human and feline tetherins inhibit the production of RD-114 [24]. Tetherin is a type II integral membrane protein consisting of an N-terminal cytoplasmic tail followed by a single transmembrane domain, followed by a coiled-coil extracellular domain important for dimerization, and a glycophosphatidyl inositol lipid anchor at its C-terminus [25]. Its antiviral mechanism is considered to involve restriction of enveloped virus release by bridging the host and virion membranes with its two opposing membrane anchors [26]. Progeny virions released from cells could also be directly tethered to each other by tetherin.

In this study, we attempted to establish the CRFK cell line with reduced risk of endogenous RD-114 by suppressing the release of infectious RD-114 from cells by the antiviral action of tetherin.

## Materials and Methods

### Establishment of CRFK cells stably expressing human tetherin

CRFK cells (ATCC CCL-94) were maintained at 37°C in a 5% CO_2_ incubator in Dulbecco's modified Eagle's medium (Sigma, St. Louis, MO) supplemented with 10% fetal bovine serum and penicillin/streptomycin. CRFK cells were transfected with an expression plasmid for human tetherin containing a FLAG-tag at the N-terminus, pTeth-FL, using Trans-IT LT-1 (Mirus Bio Corp., Madison, WI) [14]. Cells stably expressing human tetherin were selected with hygromycin (Calbiochem, San Diego, CA). Seven clones were isolated by serial dilution of hygromycin-resistant cells using 96-well plates and cultured. Tetherin expression in each cell clone was examined by Western blotting analysis using anti-FLAG M2 antibody (Sigma, St. Louis, MO) and FACS analysis using PE-labeled anti-CD317 (tetherin) antibody (BioLegend, San Diego, CA). For FACS analysis, a FACSCalibur™ flow cytometer (BD Biosciences, San Jose, CA) was used. The cell clone showing the highest level of human tetherin expression was established as the Teth-CRFK cell line.

### Quantification of RD-114 production by real-time RT-PCR

CRFK and Teth-CRFK cells (1×10^6^ cells) were cultured for 3 days. The culture media from each cell line were collected and then centrifuged to sediment RD-114 virions [24], [27]. The copy number of viral RNA derived from RD-114 virion released from each cell line into the culture media were measured by real-time RT-PCR as described previously [24], [27].

### Titration of infectious RD-114

The infectious titer of RD-114 virus produced from CRFK cells and Teth-CRFK cells was quantified using the method modified the *Lac*Z marker rescue assay established previously [28]. Briefly, CRFK and Teth-CRFK cells were transduced with the nlsLacZ gene by inoculation of *lacZ* (FeLV-B) virus [29]. Transduced cells (1×10^6^ cells), CRFK (LacZ) and Teth-CRFK (LacZ) cells, were cultured for 3 days. We confirmed by X-gal staining that the transduction efficiency of *lacZ* gene into CRFK (LacZ) and Teth-CRFK (LacZ) cells were 100%. After changing the culture media, cells were further incubated for 4 days. The culture supernatants were collected and then filtrated through 0.45-µm filters. The filtrated culture supernatants were inoculated into TE671 cells. After 2 days, cells were stained with X-gal and then foci were counted.

### MTT assay

The cell viabilities of CRFK cells and Teth-CRFK cells were measured by MTT assay using a Cell Proliferation Kit I (MTT) (Roche Applied Science, Indianapolis, IN). CRFK and Teth-CRFK cells were seeded at 1×10^5^ cells/well into 96-well plates and then cultured for 2 days. The assay was performed according to the manufacturer's instructions. The spectrophotometric absorbance of the sample was measured at 595 nm wavelength using a TriStar LB941 (Berthold Technologies, Bad Wildbad, Germany).

### Infection and titration of parvoviruses

In this study, we used two parvovirus strains, TU1 strain [30] of feline panleukopenia virus (FPLV) and V220 strain [31] of canine parvovirus 2a (CPV-2a). CRFK and Teth-CRFK cells were inoculated with TU1 strain of FPLV or V220 strain of CPV-2a at a multiplicity of infection (moi) of 0.1. After incubation at 37°C for 1 h, the cells were washed twice with medium and then cultured with 2 ml of DMEM supplemented with 10% fetal bovine serum for 2 days. The titers of parvoviruses produced in CRFK or Teth-CRFK cells were measured in TCID_50_ using FL74 cells (feline lymphoblastoid cell line) as described previously [32], [33].

## Results and Discussion

### Expression of human tetherin in Teth-CRFK cells

**Figure 1:**
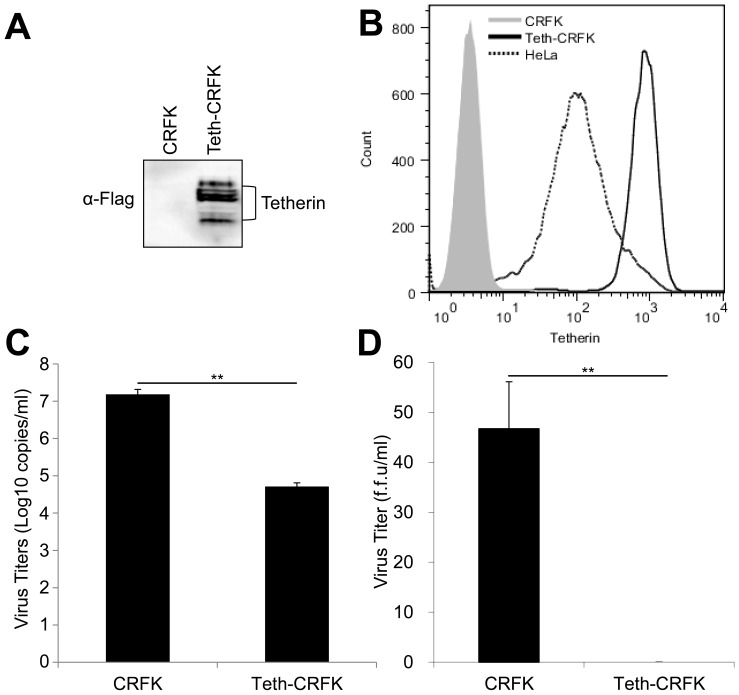
Establishment of Teth-CRFK cells and reduction of RD-114 production in Teth-CRFK cells. (A) The expression of human tetherin in Teth-CRFK cells was analyzed by Western blotting using anti-FLAG antibody. (B) CRFK, Teth-CRFK, and HeLa cells were stained with PE-labeled anti-CD317 antibody and analyzed by FACS. (C) CRFK and Teth-CRFK cells (1×106 cells/well) were cultured for 3 days. RD-114 released into the culture supernatant was quantified by real-time RT-PCR for its viral RNA using primers targeting the RD-114 pol region. Data represent means ± standard deviation of three independent experiments. (D) The productions of infectious RD-114 from CRFK and Teth-CRFK cells were quantified by LacZ marker rescue assay. Significance by Student's t test: **, P<0.01.

The CRFK cell line stably expressing human tetherin, Teth-CRFK cell line, was established as described in Materials and Methods. The expression of human tetherin in Teth-CRFK cells was confirmed by Western blotting analysis using anti-FLAG M2 antibody (Fig. 1A) and by FACS analysis using PE-labeled anti-CD317 (tetherin) antibody (Fig. 1 B). Both analyses showed distinct expression of human tetherin in Teth-CRFK cells, while the expression of tetherin was not observed in CRFK cells. Moreover, FACS analysis showed that the cell-surface expression level of tetherin in Teth-CRFK cells was much higher than that in HeLa cells, which is a human cell line constitutively expressing tetherin (Fig. 1B). The tetherin expression in Teth-CRFK cells has been maintained at high level even after more than 10 passages.

### Reduction of RD-114 release from Teth-CRFK cells

CRFK cells are known to be constitutively produce infectious RD-114 [2], [10], [11]. To confirm the inhibition of RD-114 production by tetherin in Teth-CRFK cells, we cultured CRFK and Teth-CRFK cells (1×10^6^ cells) for 3 days and measured the copy number of viral RNA derived from RD-114 virion released from each cell line into the culture media by real-time RT-PCR. As shown in Fig. 1C, RD-114 release from Teth-CRFK cells was reduced to 1/294 that from CRFK cells. We also established the cell lines stably transfected with an empty vector and measured RD-114 release. RD-114 released from these cells was similar to that from CRFK cells (data not shown).

We also quantified the infectious titer of RD-114 virus produced from CRFK cells and Teth-CRFK cells using the *Lac*Z marker rescue assay. The infectious titer of RD-114 virus produced from CRFK cells was 46.7 focus-forming units (f.f.u.)/ml, while that produced from Teth-CRFK cells was below the detection limit (<1 f.f.u./ml) (Fig. 1D). These results indicated that the production of infectious RD-114 virus in CRFK-Teth cells was markedly suppressed.

### Cell viability of Teth-CRFK cells

**Figure 2:**
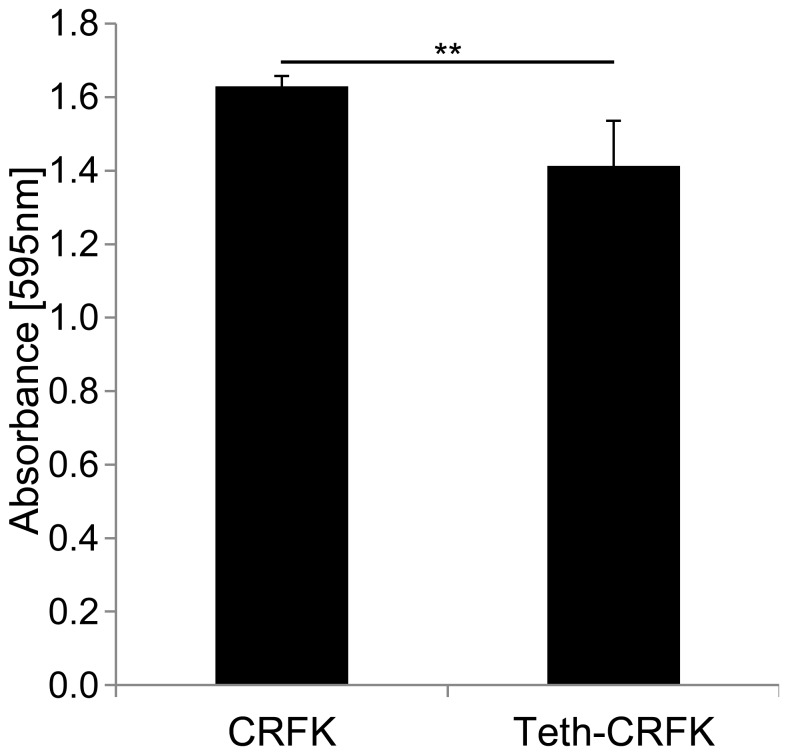
Cell viabilities of CRFK and Teth-CRFK cells. CRFK and Teth-CRFK cells were seeded at 1×105 cells/well into 96-well plates, and cultured for 2 days. Cell proliferation was analyzed by MTT assay. The spectrophotometric absorbance of the samples was measured at 595 nm. Data represent means ± standard deviation. Significance by Student's t test: **, P<0.01.

We next examined the cell viability of Teth-CRFK cells by MTT assay, since the constitutive expression of tetherin may affect the proliferation of CRFK cells. As shown in Fig. 2,viability of Teth-CRFK cells was slightly lower than that of CRFK cells.

### The growth of parvoviruses in Teth-CRFK cells

Feline cell lines, including CRFK cells, have been widely used to manufacture vaccines for parvoviruses such as feline panleukopenia virus (FPLV) and canine parvoviruses (CPVs). Indeed, there have been some reports regarding contamination of commercial live-attenuated vaccines for these viruses with infectious RD-114 [4]–[7], [34]. Tetherin is thought to inhibit the progeny virion release of a variety of enveloped viruses by directly tethering virions to cells, briefly by anchoring one end of the molecule on the cell membrane and the other end on the viral envelope. On the other hand, it is likely that tetherin cannot inhibit the production of nonenveloped viruses due to the absence of viral envelope which is the platform for its anchoring. Thus, the growth of parvoviruses is expected not to be inhibited by tetherin as parvoviruses are nonenveloped viruses.

**Figure 3:**
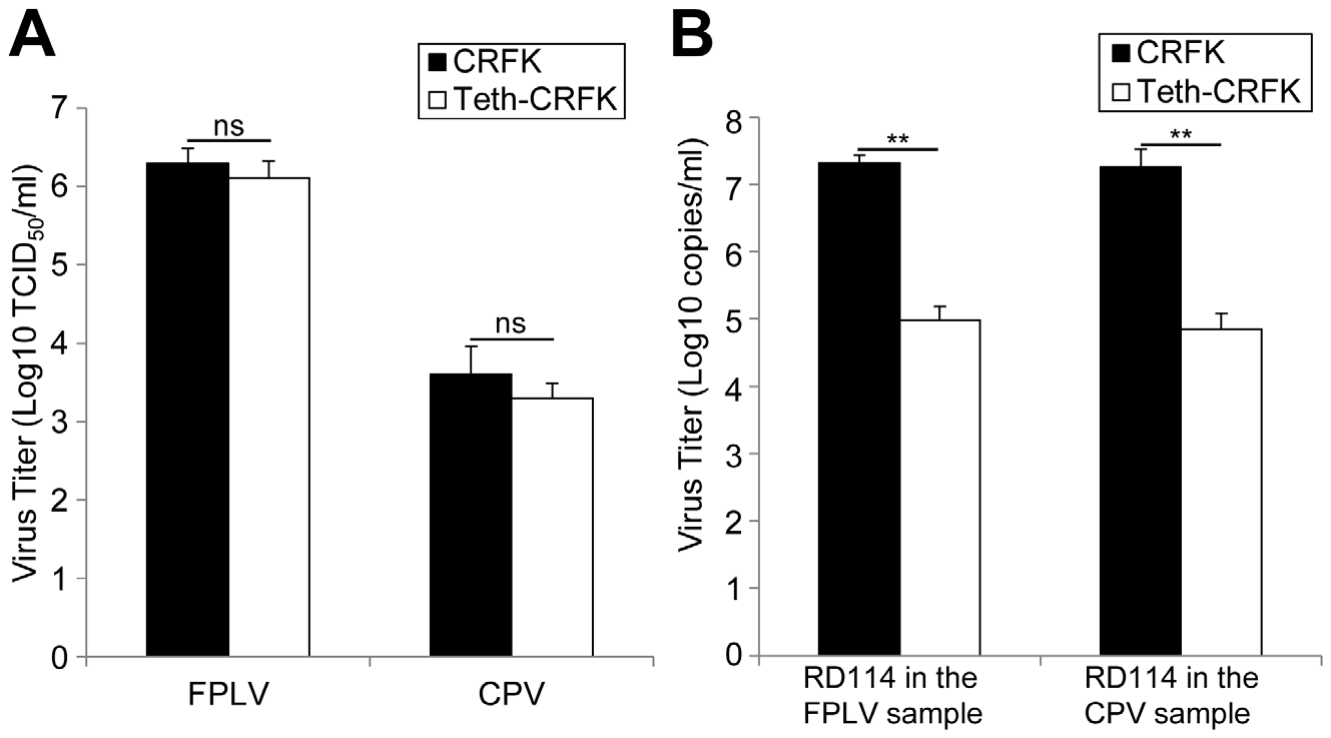
Parvovirus propagation is similar in Teth-CRFK cells and in CRFK cells. (A) CRFK and Teth-CRFK cells were inoculated with TU1 strain of FPLV or V220 strain of CPV-2a at a multiplicity of infection (moi) of 0.1. After incubation at 37°C for 1 h, the cells were washed twice with medium and then cultured with 2 ml of DMEM supplemented with 10% fetal bovine serum for 2 days. The titers of parvoviruses produced in CRFK or Teth-CRFK cells were measured in TCID50 using FL74 cells. Data represent means ± standard deviation of three independent experiments. (B) RD-114 in the samples in (A) was quantified by real-time RT-PCR as done in Fig. 1C. Data represent means ± standard deviation of three independent experiments. Significance by Student's t test: **, P<0.01; ns, non-significant.

To examine the effects of tetherin expression on the growth of parvoviruses, we next examined the growth of parvoviruses in Teth-CRFK cells. CRFK and Teth-CRFK cells were infected with TU1 strain of FPLV or V220 strain of CPV-2a and then incubated for 2 days. The titers of parvoviruses produced in CRFK or Teth-CRFK cells were measured in TCID_50_ using FL74 cells. As shown in Fig. 3A, both FPLV and CPV were propagated to a similar extent in CRFK cells and Teth-CRFK cells, although Teth-CRFK cells showed lower viability than CRFK cells (Fig. 2). This indicates that the expression of tetherin had little effect on parvovirus growth. To our knowledge, this is the first report that the expression of tetherin has little effect on parvovirus growth. It may suggest that tetherin also does not inhibit the production of other nonenveloped viruses, including caliciviruses, picornaviruses, adenoviruses, and papillomaviruses, although further analyses for these viruses would be required.

We also measured the titer of RD-114 in the parvovirus preparations from both CRFK cells and Teth-CRFK cells by real-time RT-PCR. As shown in Fig. 3B, the titer of RD-114 in the preparation of FPLV or CPV from Teth-CRFK cells was less than 1/200 of that from CRFK cells, respectively. In addition, the *Lac*Z marker rescue assay showed that infectious RD-114 was not detected in the samples from Teth-CRFK cells infected with FPLV and CPV (data not shown). These were consistent with the results from Fig. 1C and D.

We previously demonstrated that RD-114 proviral DNA was never detected in PBMC from the dogs inoculated with 1,200 FFU of RD-114 (Yoshikawa et al., unpublished observation). Thus, we estimate that parvovirus vaccines prepared from the Teth-CRFK cell line should not establish RD-114 infection in vivo even after concentrating the supernatants >1,200-fold

Taken together, these observations suggested that Teth-CRFK cells would be useful for the manufacture of live-attenuated vaccines of nonenveloped viruses or biological substances with reduced risk of contamination with infectious RD-114, although the use for enveloped viruses which could be inhibited by tetherin might be limited. Furthermore, similar strategies using tetherin would be applicable to establish various cell lines with reduced risk of endogenous retrovirus contamination.
